# Clinical, Histopathological, Dermoscopic Features, and *BRAF*, *NRAS*, and Cell Cycle Genes’ Mutation Status in Cutaneous Melanoma

**DOI:** 10.3390/cancers17162688

**Published:** 2025-08-19

**Authors:** Maria A. Pizzichetta, Jerry Polesel, Maria C. Sini, Antonella Manca, Sara Simi, Panagiotis Paliogiannis, Caterina Pinzani, Paola Corsetti, Vincenzo Canzonieri, Stefano Astorino, Paola Pasquini, Maria T. Corradin, Sandro Sulfaro, Maurizio Lombardo, Michele Cerati, Giovanna Moretti, Marisa Falduto, Giovanni B. Maestrale, Antonio Cossu, Mattia Garutti, Ignazio Stanganelli, Fabio Puglisi, Serena Bonin, Daniela Massi, Giuseppe Palmieri

**Affiliations:** 1 Department of Medical Surgical and Health Sciences, University of Trieste, Strada di Fiume 447, 34129 Trieste, Italy; vcanzonieri@cro.it (V.C.); sbonin@units.it (S.B.); 2 Department of Medical Oncology, Centro di Riferimento Oncologico di Aviano (CRO) IRCCS, via Gallini 2, 33081 Aviano, Italy; pinzca@gmail.com (C.P.); paola.corsetti@cro.it (P.C.); mattia.garutti@cro.it (M.G.); fabio.puglisi@cro.it (F.P.); 3 Unit of Cancer Epidemiology, Centro di Riferimento Oncologico di Aviano (CRO) IRCCS, via Gallini 2, 33081 Aviano, Italy; polesel@cro.it; 4 Unit of Cancer Genetics, Istituto di Ricerca Genetica e Biomedica—Consiglio Nazionale delle Ricerche (IRGB-CNR), Traversa La Crucca 3, 07100 Sassari, Italy; mariacristina.sini@cnr.it (M.C.S.); antonella.manca@cnr.it (A.M.); giovannibattista.maestrale@cnr.it (G.B.M.); giuseppe.palmieri@cnr.it (G.P.); 5 Section of Pathology, Department of Health Sciences, University of Florence, viale Pieraccini 6, 50139 Florence, Italy; sara.simi@unifi.it (S.S.); daniela.massi@unifi.it (D.M.); 6 Pathology and Histology Unit, Department of Medicine, Surgery and Pharmacy, University of Sassari, via Roma 151, 07100 Sassari, Italy; ppaliogiannis@uniss.it (P.P.); cossu@uniss.it (A.C.); 7 Division of Pathology, Centro di Riferimento Oncologico di Aviano (CRO) IRCCS, via Gallini 2, 33081 Aviano, Italy; 8 Division of Dermatology, Celio Hospital, Piazza Celimontana 50, 00184 Rome, Italy; stefano.astorino@esercito.difesa.it; 9 Dermatopathology Research Unit, San Gallicano Dermatological Institute, IRCCS, via Chianesi 53, 00144 Rome, Italy; paola.pasquini@ifo.it; 10 Dermatologic Clinic, Santa Maria degli Angeli General Hospital, via Montereale 24, 33170 Pordenone, Italy; teresa.corradin@asfo.sanita.fvg.it; 11 Consultant, Department of Pathology, University of Trieste, Strada di Fiume 447, 34129 Trieste, Italy; ssulfaro@libero.it; 12 Division of Dermatology, Varese Hospital, viale Borri 57, 21100 Varese, Italy; maurizio.lombardo@asst-settelaghi.it; 13 Division of Pathology, Varese Hospital, viale Borri 57, 21100 Varese, Italy; michele.cerati@asst-settelaghi.it; 14 Division of Dermatology, Papardo Hospital, Contrada Papardo, 98158 Messina, Italy; giovannamoretti@aopapardo.it; 15 Division of Pathology, Papardo Hospital, Contrada Papardo, 98158 Messina, Italy; marisafalduto@aopapardo.it; 16 Skin Cancer Unit, IRCCS Istituto Romagnolo per lo Studio dei Tumori “Dino Amadori” (IRST), via Maroncelli 40, 47014 Meldola, Italy; ignazio.stanganelli@irst.emr.it; 17 Department of Dermatology, University of Parma, via Gramsci 14, 43126 Parma, Italy; 18 Department of Medicine (DMED), University of Udine, via Colugna 50, 33100 Udine, Italy; 19 Immuno-Oncology and Targeted Cancer Biotherapies, Azienda Ospedaliero Universitaria di Sassari, via San Pietro 10, 07100 Sassari, Italy

**Keywords:** *BRAF*, cell cycle genes, cutaneous melanoma, dermoscopy, *NRAS*

## Abstract

**Background/Objectives**: The clinical, histopathological, and dermoscopic features may be associated with melanoma mutational status. The aims of the study were to assess the clinical, histopathological, and dermoscopic features of melanoma to identify their correlation with *BRAF*, *NRAS*, and cell cycle genes’ mutational status in melanoma. **Methods**: The clinical, histopathological information, dermoscopic images, and genomic DNA of 55 histopathologically diagnosed primary cutaneous melanomas were retrospectively analyzed. Next-generation sequencing (NGS) assays were conducted on the Ion GeneStudio S5 platform (Thermo Fisher Scientific, Waltham, MA, USA), using the Ion AmpliSeq™ Italian Melanoma Intergroup Somatic Panel. **Results**: Overall, 55 melanomas, including 30 superficial spreading, 24 nodular, and 1 naevoid, were analyzed. *BRAF* mutation was more frequently observed in ulcerated melanoma (16/23; 69.6%), with mitotic rate ≥ 5 n/mm^2^ (8/11; 72.7%), while *NRAS* mutation was more common in amelanotic/hypomelanotic (8/17; 70.0%) and nodular melanoma (10/24; 41.7%). Dermoscopically, shiny white structures (OR = 3.50; 95% confidence interval: 1.13–10.84) were associated with *BRAF*-mutated melanomas, whereas a homogeneous disorganized pattern was associated with *NRAS*-mutated melanomas (OR = 6.96; 1.49–32.53). The risk of diagnosing cell cycle gene-mutated melanomas was significantly increased in presence of vascular patterns (OR = 4.50; 1.33–15.20), linear irregular (OR = 3.75; 1.18–11.92), polymorphous vessels (OR = 4.05; 1.27–12.97), and milky red globules/areas (OR = 3.14; 1.00–9.89). The blue-white veil was significantly associated with P53 mutation (OR = 35.84; 2.01–640.2). **Conclusions**: Conversely to *Wild Type*, *BRAF*, *NRAS*, and cell cycle gene-mutated melanomas were significantly associated with clinical and dermoscopic features underlying a more aggressive melanoma phenotype. The vascular pattern, linear irregular, polymorphous vessels, and milky-red globules/areas may be considered predictors of cell cycle mutated melanomas.

## Simple Summary

In this study cutaneous melanomas were evaluated to identify the correlation between clinical, histopathological, dermoscopic features, and *BRAF*, *NRAS*, and cell cycle genes’ mutational status. *BRAF* mutation was more frequently observed in ulcerated melanomas with a high mitotic rate ≥ 5 n/mm^2^ (a measure of how fast melanoma cells are growing), while NRAS mutation was associated with amelanotic/hypomelanotic (subtype with little or no pigmentation) and nodular melanoma. The risk of diagnosing cell cycle gene-mutated melanomas was significantly increased in presence of vascular patterns, linear irregular, polymorphous vessels, and milky red globules/areas (vessel types seen in melanomas). Our findings suggest that, conversely to non-mutated melanomas, *BRAF*, *NRAS*, and cell cycle gene-mutated melanomas were significantly associated with clinical, histopathological, and dermoscopic characteristics underlying a more aggressive melanoma phenotype. The potential clinical relevance of this study is that our findings might identify melanoma cases that could respond to anti-angiogenic therapies.

## 1. Introduction

The clinical, histopathological, and dermoscopic features of melanoma may be associated with melanoma mutation status [1]. *BRAF* mutation is more frequently found in superficial spreading melanoma/low-cumulative sun damage (Low-CSD) melanomas arising on non-chronic sun-damaged skin; differently, *NRAS* mutation is more frequent in nodular type and melanoma arising on chronic sun-damaged skin [2]. Somatic mutations in the oncogenes *BRAF* and *NRAS* result in activation of the intracellular mitogen-activated protein kinase (MAPK) pathway that is involved in melanoma pathogenesis [3].

The *BRAF* oncogene encodes a serine–threonine kinase, a key component of the MAPK signaling pathway, leading to increased cell proliferation and survival because of overactivation of the MAPK pathway [3]. Somatic mutations in the V600 codon of *BRAF* have been found in 35–50% of melanomas and the amino acid substitution from a valine to a glutamic acid at codon 600 (V600E) accounts for 74–92% of *BRAF* mutations in melanoma [4]. The somatic mutation in the *NRAS* oncogene was reported in 28% of primary and metastatic melanoma; *NRAS* encodes GTP-binding proteins located upstream of the *BRAF* within the MAPK pathway [4].

The somatic mutations in the cell cycle genes, such as *CCND1*, *CDKN2A*, *CDK4*, and *TP53* that play a crucial role in cell cycle control and tumor suppression, may also be involved in melanoma pathogenesis [3,4]. *CCND1* and *CDK4*, key cell cycle regulators, are often overexpressed in melanomas, leading to uncontrolled cell proliferation [3]. The *CDKN2A* acts as a tumour suppressor that regulate the cell cycle and its inactivation, commonly observed in melanomas, results in unchecked cell cycle progression [3]. The *TP53*, another tumour suppressor gene, is responsible for DNA damage response control and apoptosis regulation [3]. Mutations in these genes are linked to melanoma initiation and progression, demonstrating the intricate balance between cell cycle proteins and melanoma pathogenesis.

In this retrospective multicenter study we investigated the association between selected genes’ mutational status and clinical, histopathological, and dermoscopic features in primary cutaneous melanomas, focusing on *BRAF*, *NRAS*, and cell cycle genes such as *CCND1*, *CDKN2A*, *CDK4*, and *TP53*. This study may have a great prognostic significance, identifying potentially “high-risk” melanoma on dermoscopic evaluation, with relevant clinical implications. This may be useful in deciding the type of follow-up that is provided as well as in identifying melanoma cases that could respond to anti-angiogenic therapies or treatment aimed at modifying the immunogenic status of the tumor microenvironment.

## 2. Materials and Methods

We collected 55 cases of histopathologically confirmed primary cutaneous melanoma from five Italian Melanoma Intergroup centers between May 2003 and January 2017. This case series included 30 superficial spreading melanoma (SSM)/Low-CSD melanoma, 24 nodular melanoma (NM, *n* = 24), and one nevoid melanoma. Each center provided clinical (i.e., gender, age at diagnosis, melanoma site) and conventional histopathological data (i.e., histotype, Breslow thickness in mm, ulceration, mitotic rate [n/mm^2^], tumor infiltrating lymphocytes [TILs], regression, sentinel lymph node) together with dermoscopic images. All centers provided representative formalin-fixed-paraffin-embedded (FFPE) tissue samples, retrieved from pathological archives of the institutions participating in the study. By January 2023, all data from the five centers were merged into a database at the Unit of Cancer Epidemiology of the Centro di Riferimento Oncologico di Aviano (Italy). In agreement with privacy regulations, data were pseudonymized using a new identification number linking patient clinical data and dermoscopic images.

All dermoscopic images were randomly sorted and evaluated by a panel of three blinded dermatologists, experts in dermoscopy, to assess the presence or absence of specific dermoscopic criteria using a standardized form (Table S1). Lesions were assessed using dermoscopic features associated with melanoma [5,6] and a consensus was met when 3/3 or 2/3 dermatologists agreed.

The study was approved by the Ethics Committee of Friuli Venezia Giulia (protocol code CEUR-Sper-082-CRO approved on 11 October 2017).

### 2.1. Mutation Analysis

Genomic DNA was isolated from representative FFPE tissue samples by GeneRead DNA FFPE Kit (QIAGEN, Hilden, Germany), which was based on particular lytic conditions to overcome inhibitory effects caused by formalin deamination of nucleic acids. For some SSM samples, only a limited amount of good-quality genomic DNA was isolated.

Next-generation sequencing (NGS) assays were conducted on the Ion GeneStudio S5 platform (Thermo Fisher Scientific, Waltham, MA, USA), using the Ion AmpliSeq™ IMI Somatic Panel, which was constituted by a highly multiplexed target selection of 25 genes involved in CM pathogenesis. Isolated genomic DNA and corresponding libraries were accurately quantified before sequence runs using quantification methods, such as Nanodrop 2000 and Qubit dsDNA HS spectrophotometers (Invitrogen, Life Technologies, Carlsbad, CA, USA).

The targeted sequencing panel (343 amplicons, with a coverage range of 125–175 bp) was designed to encompass the entire coding sequencing of five genes (*CDKN2A*, *ARID2*, *BAP1*, *CCND1*, *MAP2K1*) and the activating mutations into the exons mainly involved in tumorigenesis for twenty other candidate driver genes (*BRAF*, *K-/H-/N-RAS*, *KIT*, *CDK4*, *TP53*, *PTEN*, *PIK3CA*, *RB1*, *MET*, *MITF*, *NF1*, *GNAQ*, *GNA11*, *ERBB4*, *NOTCH1*, *KDR*, *DDX3X*, *PPP6C*). Just as an example, the gene regions included in the NGS-based analysis were the exons 11 and 15 for *BRAF*, the exons 1, 3–7, and 9 for *TP53*, and the exons 1, 3, 5–8 for *PTEN*. The complete list of gene exons covered by our NGS panel was reported in a previous report [7]. Total coverage of all targeted loci was ≥100 reads per amplicon and 5% allele frequency. The cell cycle genes analyzed in this study were *CCND1*, *CDKN2A*, *CDK4*, and *TP53.*

The Torrent Suite software version 5.12.3 (Thermo Fisher Scientific, Waltham, MA, USA) was used to analyze and classify the raw signal data after a successful sequencing reaction. Variant calls were further analyzed by the Ion Reporter™ Software (version 5.20) using a customized panel analysis workflow that allows for variant filtering and annotation using COSMIC version 92, Single Nucleotide Polymorphism Database build 151, PolyPhen-2 and SIFT, and Varsome (www.varsome.com; accessed on 31 July 2025). Amino acid predictions were carried out using in silico prediction algorithms SIFT and PolyPhen-2 to predict potential deleterious effects on protein function. The clinical significance of all identified variants was examined using the standards and guidelines for the interpretation of sequence variants recommended by the American College of Medical Genetics and Genomics (Laboratory Quality Assurance Committee) and the Association for Molecular Pathology.

### 2.2. Statistical Analysis

Clinical, histopathological, and dermoscopic features were reported as absolute frequencies and percentages; differences in the distribution of these features across mutational status were evaluated through Fisher’s exact test. The association of dermoscopic features with mutational status was evaluated through odds ratio (OR), with a corresponding 95% confidence interval (CI), calculated by means of an unconditional logistic regression model. Considering the small sample size, only univariate models were considered. Although statistical significance was claimed only for *p* < 0.05, in consideration of the small sample size, results with *p* < 0.10 were also reported as potential associations.

## 3. Results

The study included 55 primary cutaneous melanomas from 55 patients (27 men, 28 women) with a median age of 54 years (range 27–81 years). Clinically, 37 melanomas were pigmented, whereas 17 cases were amelanotic/hypomelanotic melanoma (AHM); datum was missing in one case. The anatomical sites were the trunk (*n* = 26), lower limbs (*n* = 15), upper limbs (*n* = 10), and other specified subsites (*n* = 4).

Table 1 shows the clinical and histopathological features according to mutational status in selected genes: *BRAF* mutation was reported in 25 cases (45.5%), *NRAS* in 12 cases (21.8%), and mutation in cell cycle genes in 20 cases (36.4%); 12 melanomas were *wild type* (*WT*) for all genes in the panel. Detailed gene mutational status of each patient is reported in Table S2. *WT* status for all genes was reported more frequently among pigmented than AHM (32.4% and 0.0%, respectively; *p* = 0.011), among SSM than nodular melanomas (33.3% and 8.3%; *p* = 0.046), in Breslow thickness ≤ 1 mm than >4 mm (40.0% and 0.0%; *p* = 0.028), non-ulcerated than ulcerated melanoma (34.4% and 4.4%; *p* = 0.007), and in melanomas with a mitotic rate < 2 than ≥5 n/mm^2^ (40.7% and 9.1%; *p* = 0.003). Conversely, *BRAF* mutation was more frequently observed in ulcerated than non-ulcerated melanomas (69.6% and 28.1%; *p* = 0.003) and in melanomas with mitotic rate ≥ 5 than <2 n/mm^2^ (72.7% and 22.2%; *p* = 0.003). By contrast, *NRAS* mutation was associated with AHM (40.0% versus 10.8% in pigmented melanoma; *p* = 0.005) and with NM (41.7% versus 3.3% in SSM; *p* = 0.001). No differences in the prevalence of *BRAF*, *NRAS*, and cell cycle gene mutations were found according to sex, age, and anatomical sites.

Table 2 shows the univariate associations between the dermoscopic features and gene mutational status, which were statistically significant at *p* < 0.10. *BRAF*-mutated melanoma was significantly associated with the presence of shiny white structures (OR = 3.50, 95% CI: 1.13–10.84; *p* = 0.030). Conversely, a homogeneous disorganized pattern was significantly associated with increased risk of *NRAS* mutated melanoma (OR = 6.96, 95% CI: 1.49–32.53; *p* = 0.014), whereas a multicomponent pattern was significantly associated with a reduced risk of *NRAS* mutated melanoma (OR = 0.16, 95% CI: 0.03–0.83; *p* = 0.029).

The risk of harboring a mutation in at least one of the cell cycle genes was significantly increased in the presence of vascular pattern (OR = 4.50, 95% CI: 1.33–15.20; *p* = 0.015), linear irregular vessels (OR = 3.75, 95% CI: 1.18–11.92), polymorphous vessels (OR = 4.05, 95% CI: 1.27–12.97), and milky red globules/areas (OR = 3.14, 95% CI: 1.00–9.89; *p* = 0.050). *TP53* was the most frequently mutated gene within the cell cycle genes, reported in 11 out of 20 patients with mutations in cell cycle genes. Interestingly, *TP53* mutation was significantly associated with the presence of the blue-white veil (OR = 35.84, 95% CI: 2.01–640.2; *p* = 0.004).

## 4. Discussion

The most striking results of our study were that *BRAF* and *NRAS* mutated melanomas were significantly associated with conventional poor prognostic variables than *WT* melanomas, such as ulceration, higher mitotic rate, NM histotype, and AHM subtype [8,9,10,11]. In line with previous reports, we found that the histological ulceration—the third most powerful indicator of survival after melanoma thickness and mitotic activity [8,9]—was significantly more frequent in *BRAF*-mutated than in melanoma with other genes mutated or *wild type* [12,13]. Furthermore, in our study, *BRAF*-mutated melanomas tended to have higher Breslow thickness and higher mitotic rate.

**Figure 1:**
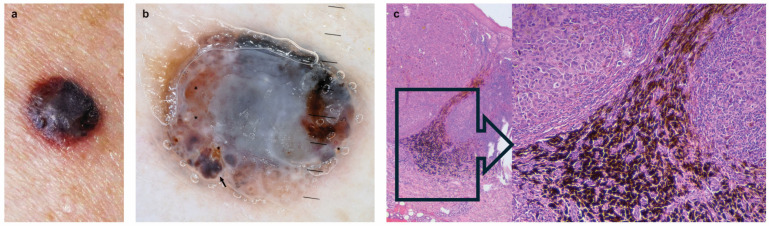
*NRAS* and *TP53* mutated nodular melanoma (NM), 2.3 mm-thick, mitotic rate ≥ 1 n/mm^2^ on the left arm of a 60-year-old woman. (**a**) Clinical image shows a black-blue reddish symmetrical nodule. (**b**) At dermoscopic examination, the lesion was typified by an overall homogeneous disorganized pattern consisting of blue-white veil, areas of black and blue pigmentation (small black arrow), and atypical vessels (black stars) (10× magnification). (**c**) Histologically, nested melanophages at the base and between dermal aggregates of atypical melanocytes under a slightly thickened epidermis (hematoxylin-eosin, 5× magnification). Inset, detail of nested melanophages (25× magnification).

**Figure 2:**
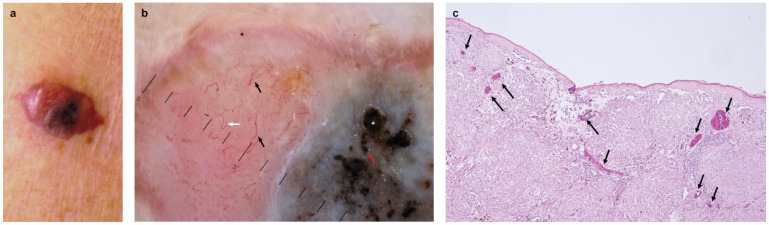
*NRAS-* and cell cycle (*CCND1* gene)-mutated melanoma, 4.9 mm-thick, and mitotic rate/mm^2^ = 11, on the forearm of an 80-year-old woman. (**a**) In the clinical image (inset) a blue-reddish plaque can be observed. (**b**) Magnified detail of polymorphous vascular pattern at dermoscopic examination characterized by serpentine (small black arrow), hairpin vessels (small white arrow), and milky-red areas (black star). In addition, black globules (small red arrow) and blotches (white star) can be observed (10× magnification). (**c**) Histologically, dilated capillaries (arrows) beneath the epidermis and between tumor dermal aggregates can be seen (hematoxylin-eosin, 10× magnification).

In agreement with Lee et al. [2], we found a significantly greater frequency of NM histotype in *NRAS*-mutated melanomas (Figure 1). In addition, we found that *NRAS*-mutated melanomas were significantly associated with AHM subtype (Figure 2). Some authors have reported that five-year survival was worse in NM than in SSM and in AHM compared to pigmented melanoma [10,11]. The poorer prognosis in patients with AHM was probably related to higher tumor stage at diagnosis, since AHM was more likely to be misdiagnosed than pigmented melanoma [11]. Interestingly, in our study, *WT* melanomas were significantly associated with pigmented, thinner, and non-ulcerated SSM. Therefore, we underline the association of *BRAF*- and *NRAS*-mutated melanomas with clinicopathologic features underlying a more aggressive melanoma phenotype.

Furthermore, the present study highlights associations between the prevalence of dermoscopic features and melanoma mutational status. Firstly, the homogeneous disorganized pattern was directly significantly associated with *NRAS*-mutated melanoma (Figure 1), whereas the multicomponent pattern was inversely associated with *NRAS*-mutated melanoma. These results are in agreement with previous reports that found the homogeneous disorganized pattern was related to NM, while the multicomponent pattern was associated with SSM [14,15,16].

In *BRAF*-mutated melanomas, we also found a significantly higher frequency of shiny white structures that reflect de novo synthesis or remodeling of collagen in the papillary dermis as a response to melanoma of the fibroblast and stroma [17,18]. Therefore, shiny white lines may be the expression of histologic regression changes, suggesting the role of *BRAF* in immunopathogenesis [12].

Interestingly, we found a significantly greater frequency of the blue-whitish veil in *TP53*-mutated melanoma (Figure 1). The presence of a blue-white veil has been highly correlated with thicker, ulcerated melanomas and a mitotic rate ≥ 1 n/mm^2^ [19,20]. From a histopathological point of view, the blue-whitish veil feature corresponds to nests of pigmented atypical melanocytes in the dermis under a thickened epidermis, reflecting the higher proliferative activity of these melanomas. Moreover, NM may exhibit dermal melanophages, usually associated with a moderate degree of inflammation, dermoscopically correlated with areas of blue pigmentation (Figure 1).

The association of dermoscopic features with mutation in cell cycle genes is a new and innovative finding of the present study. Indeed, such mutations were significantly associated with the presence of vascular pattern, linear irregular, polymorphous vessels, and milky-red globules/areas; dilated capillaries beneath the epidermis and between dermal aggregates of atypical melanocytes can be seen in Figure 2. Neoangiogenesis is the formation of new blood vessels from pre-existing “quiescent” vasculatures, occurring during tumor growth in the presence of pro-angiogenic factors secreted by melanoma and/or as the reaction to hypoxia [21,22]. The rapid proliferation of melanocytes separates cells from the vasculature, leading to increasing demands of oxygen and nutrient demands that require new vessels to support the continued tumor growth [23]. Hypoxia may activate Hypoxia-Inducible Factor-1 alpha (HIF-1α), encoded by the *HIF-1A* gene. HIF-1α is a regulator of O_2_ homeostasis and an inducer of various pro-angiogenic factors, the most important of which is vascular endothelial growth factor-A (VEFG-A) [23,24]. In conditions of hypoxia, HIF-1α is translocated into the nucleus and activates the expression of genes involved in angiogenesis together with genes involved in metabolism, cellular proliferation, and metastasis, leading to melanoma progression [23,24]. The hypoxia status and the loss of the tumor-suppressive function of p53 are two common biological events in solid tumors [25].

Neoangiogenesis plays an essential role in development and growth of melanoma; a high-density vascular network is associated with the evolution of atypical intraepidermal melanocytic to melanoma in situ [26]. From a dermoscopic point of view, the increased vascular volume appears as milky-red areas and/or vascular structures that may be linked with melanoma thickness [27,28]. The presence of vascular pattern, linear irregular vessels, polymorphous vessels, and milky-red globules/area—predictors of cell cycle genes mutated melanomas—is suggestive of intermediate-thick melanomas [28,29]. Moreover, these vessels and blue-whitish veil were closely related to the neoplastic proliferation of cell cycle mutated melanomas and they were found to be associated with thicker lesions and with the risk of melanoma recurrence [19,20,30]. Conversely to *WT*, cell cycle genes and *TP53* mutated melanomas were significantly associated with dermoscopic features underlying a more aggressive phenotype. Furthermore, dermoscopy may also have an important prognostic significance in predicting melanoma with high metastatic potential. Indeed, the blue-whitish veil, milky-red areas, and low pigmentation found in amelanotic melanoma were considered prognostic criteria for distant metastasis [31,32,33,34].

The small sample size is to be acknowledged among study limitations since it impacts study power and the possibility of conducting multivariable analyses. However, the availability of mutational status together with a comprehensive dermoscopic profile is challenging, limiting the sample size of similar studies.

## 5. Conclusions

In synthesis, we found that increased vascularization, linear irregular vessels, polymorphous vessels, and milky-red globules/areas may be considered predictors of mutations in cell cycle genes. Conversely to *WT* melanomas, those harboring a mutation in *BRAF*, *NRAS*, or cell cycle reported clinical and dermoscopic features underlying a more aggressive phenotype. Despite the small size of our study being a limitation, these findings may be of considerable prognostic impact, somehow predicting the disease behavior of the different melanoma “types” and identifying patients at greater risk of relapse. This could be useful in tailoring follow-up programmes. In addition, these findings might identify melanoma cases that could respond to anti-angiogenic therapies or treatments aimed at modifying the immunogenic status of the tumor microenvironment.

## Figures and Tables

**Table 1:** Sociodemographic and clinical characteristics according to mutational status.

Characteristics	All Patients	Mutated Gene							
		*BRAF*		*NRAS*		Cell Cycle		*Wild Type* All Genes	
	*n*	*n*	(%)	*n*	(%)	*n*	(%)	*n*	(%)
All	55	25	(45.5)	12	(21.8)	20	(36.4)	12	(21.8)
Gender									
Male	27	12	(44.4)	3	(11.1)	10	(37.0)	7	(25.9)
Female	28	13	(46.4)	9	(32.1)	10	(35.7)	5	(17.9)
Fisher’s exact test		*p* = 1.000		*p* = 0.101		*p* = 1.000		*p* = 0.528	
Age (years)									
<60	34	15	(44.1)	5	(14.7)	12	(35.3)	9	(26.5)
≥60	21	10	(47.6)	7	(33.3)	8	(38.1)	3	(14.3)
Fisher’s exact test		*p* = 1.000		*p* = 0.178		*p* = 1.000		*p* = 0.337	
Site									
Trunk and back	26	11	(42.3)	4	(15.4)	11	(42.3)	7	(29.9)
Lower limbs	15	7	(46.7)	3	(20.0)	3	(20.0)	4	(26.7)
Upper limbs	10	5	(50.0)	5	(50.0)	5	(50.0)	0	(0.0)
Other	4	2	(50.0)	0	(0.0)	1	(25.0)	1	(25.0)
Fisher’s exact test		*p* = 0.975		*p* = 0.127		*p* = 0.358		*p* = 0.271	
Melanoma type ^a^									
Pigmented	37	14	(37.8)	4	(10.8)	11	(29.7)	12	(32.4)
Amelanotic/hypomelanotic	17	10	(70.0)	8	(40.0)	8	(47.1)	0	(0.0)
Fisher’s exact test		*p* = 0.238		*p* = 0.005		*p* = 0.237		*p* = 0.011	
Histological type ^b^									
Superficial spreading	30	12	(40.0)	1	(3.3)	8	(26.7)	10	(33.3)
Nodular	24	13	(54.2)	10	(41.7)	11	(45.8)	2	(8.3)
Fisher’s exact test		*p* = 0.411		*p* = 0.001		*p* = 0.164		*p* = 0.046	
Breslow thickness (mm)									
≤1	25	7	(28.0)	2	(8.0)	7	(28.0)	10	(40.0)
>1 to 2	7	3	(43.9)	3	(42.9)	3	(42.9)	1	(14.3)
<2 to 4	15	9	(60.0)	5	(33.3)	7	(46.7)	1	(6.7)
>4	8	6	(75.0)	2	(25.0)	3	(37.5)	0	(0.0)
Fisher’s exact test		*p* = 0.063		*p* = 0.080		*p* = 0.657		*p* = 0.028	
Histologic ulceration									
Absent	32	9	(28.1)	9	(28.1)	12	(37.5)	11	(34.4)
Present	23	16	(69.6)	3	(13.0)	8	(34.8)	1	(4.4)
Fisher’s exact test		*p* = 0.003		*p* = 0.321		*p* = 1.000		*p* = 0.007	
Histologic regression									
Absent	50	23	(46.0)	12	(24.0)	19	(38.0)	10	(20.0)
Present	5	2	(40.0)	0	(0.0)	1	(20.0)	2	(40.0)
Fisher’s exact test		*p* = 1.000		*p* = 0.574		*p* = 0.643		*p* = 0.298	
Mitotic rate (n/mm^2^) ^a^									
<2	27	6	(22.2)	4	(14.8)	7	(25.9)	11	(40.7)
2 to <5	16	10	(62.5)	6	(37.5)	9	(56.3)	0	(0.0)
≥5	11	8	(72.7)	2	(18.2)	3	(27.3)	1	(9.1)
Fisher’s exact test		*p* = 0.003		*p* = 0.255		*p* = 0.140		*p* = 0.003	
Tumor infiltrating lymphocytes (TILs)									
Absent	10	7	(70.0)	2	(20.0)	4	(40.0)	0	(0.0)
Brisk	11	4	(36.4)	2	(18.2)	6	(54.6)	3	(27.3)
Non-brisk	34	14	(41.2)	8	(23.5)	10	(29.4)	9	(26.5)
Fisher’s exact test		*p* = 0.253		*p* = 1.000		*p* = 0.301		*p* = 0.187	
Solar elastosis ^a^									
Absent	27	10	(37.0)	7	(25.9)	10	(37.0)	6	(22.2)
Present	23	13	(56.5)	4	(17.4)	8	(34.8)	5	(21.7)
Fisher’s exact test		*p* = 0.255		*p* = 0.515		*p* = 1.000		*p* = 1.000	
Sentinel lymph node ^a^									
Negative	20	10	(50.0)	6	(30.0)	9	(45.0)	3	(15.0)
Positive	13	9	(69.2)	3	(23.1)	5	(38.5)	1	(7.7)
Not performed	19	5	(26.3)	3	(15.8)	3	(15.8)	8	(42.1)
Fisher’s exact test		*p* = 0.061		*p* = 0.649		*p* = 0.139		*p* = 0.069	

^a^ The sum does not add up to total because of missing values. ^b^ One patient reported nevoid melanoma.

**Table 2:** Dermoscopic features according to mutational status in selected genes.

Gene Dermoscopic Feature	Unmutated		Mutated		OR (95% CI) ^a^	χ^2^ Test
	*n*	(%)	*n*	(%)		
***BRAF***	**30**		25			
White color	14	(46.7)	18	(72.0)	2.94 (0.95–9.10)	*p* = 0.062
Brown dots/globules	21	(75.0)	12	(50.0)	0.33 (0.10–1.08)	*p* = 0.067
Blue-white veil	16	(53.3)	19	(76.0)	2.77 (0.86–8.88)	*p* = 0.086
Shiny white structures	8	(26.7)	14	(56.0)	3.50 (1.13–10.84)	*p* = 0.030
Dotted vascular pattern	4	(13.3)	9	(36.0)	3.66 (0.97–13.85)	*p* = 0.057
Arborizing vascular pattern	1	(3.3)	5	(20.0)	7.25 (0.79–66.83)	*p* = 0.081
Polymorphous vascular pattern	10	(33.3)	14	(56.0)	2.55 (0.85–7.61)	*p* = 0.095
***NRAS***	43		12			
Black color	34	(79.1)	6	(50.0)	0.27 (0.07–1.02)	*p* = 0.054
Homogeneous disorganized	4	(9.2)	5	(41.7)	6.96 (1.49–32.53)	*p* = 0.014
Multicomponent	25	(58.1)	2	(18.2)	0.16 (0.03–0.83)	*p* = 0.029
Pigment network	16	(37.2)	1	(8.3)	0.39 (0.13–1.14)	*p* = 0.086
Streaks/pseudopods	18	(41.9)	1	(8.3)	0.35 (0.12–1.05)	*p* = 0.062
**Cell cycle genes**	35		20			
Blue-white veil	19	(54.3)	16	(80.0)	3.37 (0.94–12.14)	*p* = 0.063
Presence of vascular pattern	14	(40.0)	15	(75.0)	4.50 (1.33–15.20)	*p* = 0.015
Linear irregular vessels	10	(28.6)	12	(60.0)	3.75 (1.18–11.92)	*p* = 0.025
Polymorphous vascular pattern	11	(31.4)	13	(65.0)	4.05 (1.27–12.97)	*p* = 0.018
Milky-red globules/areas	13	(34.1)	13	(65.0)	3.14 (1.00–9.89)	*p* = 0.050
***TP53***	44		11			
Blue color	24	(54.6)	10	(90.9)	8.33 (0.98–70.79)	*p* = 0.052
Black dots/globules	21	(51.2)	9	(81.8)	4.29 (0.82–22.31)	*p* = 0.084
Blue-white veil	24	(54.6)	11	(100)	35.84 (2.01–640.2)	*p* = 0.004
Linear irregular vessels	15	(34.1)		(63.6)	3.38 (0.85–13.42)	*p* = 0.083

^a^ Odds ratio (OR) of being mutated versus unmutated, with corresponding 95% confidence interval (CI), were estimated for each gene from unconditional univariate logistic regression model.

## Data Availability

The datasets generated and/or analyzed during the current study are available from the corresponding author on reasonable request.

## Supplementary Materials

The following supporting information can be downloaded at https://www.mdpi.com/article/10.3390/cancers17162688/s1, Table S1: Form for dermoscopic evaluation. Table S2: Individual gene mutational status of study patients.

## Abbreviations

The following abbreviations are used in this manuscript:

AHM	Amelanotic/hypomelanotic melanoma
CI	Confidence interval
CSD	Cumulative sun damage
FFPE	Formalin-fixed-paraffin-embedded
HIF-1α	Hypoxia-Inducible Factor-1 alpha
MAPK	Mitogen-activated protein kinase
NGS	Next-generation sequencing
NM	Nodular melanoma
OR	Odds ratio
SSM	Superficial spreading melanoma
TILs	Tumor-infiltrating lymphocytes
VEGF-A	Vascular endothelial growth factor-A
*WT*	*Wild-type*
