# Supplementary material for: Clinical, Histopathological, Dermoscopic Features, and BRAF, NRAS, and Cell Cycle Genes’ Mutation Status in Cutaneous Melanoma
Source: Cancers (Basel). 2025 Aug 19;17(16):2688. doi: 10.3390/cancers17162688 (PMC12384477; doi:10.3390/cancers17162688)
Supplement: Supplementary file 1 [file cancers-17-02688-s001.zip › Table S1.pdf]

Table S1. Form for dermoscopic evaluation

CARAMEL STUDY

ID-PAZIENTE: File N° ....

Age (years) |__|__| **SEX** |1| M |2| W

Site: |1| Head/neck |2| Trunk |3| Back |4| Upper limbs |5|Lower limbs
|6|Acral |7| Scalp |8| Other: ....

DERMOSCOPY

Dermoscopy.. |1| Polarized |2| Non Polarized
Lesion.. |1| Melanocytic |2| Non-Melanocytic
Shape .... |1| Symmetric |2| Asymmetric
Pigmentation pattern .... |1| Symmetric |2| Asymmetric
Distribution of structures .... |1| Symmetric |2| Asymmetric
Colors: White.. |1| Absent |2| Present
Yellow... |1| Absent |2| Present
Pink... |1| Absent |2| Present
Red .... |1| Absent |2| Present
Light brown .... |1| Absent |2| Present
Dark brown................................... |1| Absent |2| Present
Grey .... |1| Absent |2| Present
Blue .... |1| Absent |2| Present
Black .... |1| Absent |2| Present
Colors:... |1| Pigmented |2|Amelanotic

Pigment pattern

Reticular.. |1| Absent |2| Present
Globular .... |1| Absent |2| Present
Homogeneous.. |1| Absent |2| Present
Globular-reticular.. |1| Absent |2| Present
Globular-homogeneous .... |1| Absent |2| Present
Reticular-homogeneous................................... |1| Absent |2| Present
Cobblestone .... |1| Absent |2| Present
Starburst .... |1| Absent |2| Present
Multicomponent .... |1| Absent |2| Present
Unspecific.. |1| Absent |2| Present

Local Features

Pigment Network .... |1| Absent |2| Regular |3| Irregular
Negative pigment network .... |1| Absent |2| Center |3|Periphery
|4| Throughout lesion
Pseudonetwork (around hair follicles).............. |1| Absent |2| Present
Angulated Lines.. |1| Absent |2| Present
Dots/Globules .... |1| Absent |2| Regular |3| Irregular
Dots/Globules color: Brown .... |1| Absent |2| Present
Grey .... |1| Absent |2| Present
Black .... |1| Absent |2| Present

Streaks/Pseudopods	|1| Absent	|2| Regular	|3| Irregular
Blotches	|1| Absent	|2| Regular	|3| Irregular
Blotches color: Brown	|1| Absent	|2| Present	
Black	|1| Absent	|2| Present	
Blue-white veil	|1| Absent	|2| Present	
Shiny white blotches/strands	|1| Absent	|2| Present	
Shiny white streaks	|1| Absent	|2| Present	
Peppering	|1| Absent	|2| Present	
White scar-like areas	|1| Absent	|2| Present	
Peripheral light brown structureless areas	|1| Absent	|2| Present	
Hypopigmented structureless areas	|1| Absent	|2| Present	
Irregular blue structureless areas	|1| Absent	|2| Present	
Blue-black pigmented areas	|1| Absent	|2| Present	
Irregular hyperpigmented areas	|1| Absent	|2| Present	
Prominent skin marking	|1| Absent	|2| Present	
Scale	|1| Absent	|2| Present	
Ulceration	|1| Absent	|2| Present	
Fingerprint-like structures	|1| Absent	|2| Present	
Sharply demarcated border	|1| Absent	|2| Present	
Moth-eaten border	|1| Absent	|2| Present	
Milia-like cyst	|1| Absent	|2| Present	
Comedo-like opening	|1| Absent	|2| Present	

Vascular patterns

Vascular pattern	|1| Absent	|2| Present
Comma	|1| Absent	|2| Present
Dotted	|1| Absent	|2| Present
Serpentine	|1| Absent	|2| Present
Linear wavy vessels	|1| Absent	|2| Present
Hairpin	|1| Absent	|2| Present
Arborizing	|1| Absent	|2| Present
Linear elical vessels	|1| Absent	|2| Present
Polymorphous	|1| Absent	|2| Present
Glomerular	|1| Absent	|2| Present
Milky-red globules/areas	|1| Absent	|2| Present
Increased vascular density	|1| Absent	|2| Present
Red rhomboidal structures	|1| Absent	|2| Present
Red pseudonetwork	|1| Absent	|2| Present
Homogeneous red areas	|1| Absent	|2| Present

Dermoscopic diagnosis

|1| Solar Lentigo |2| Common Melanocytic nevus |3| Seborrheic keratosis

|4| Lentigo maligna |5| Atypical melanocytic nevus |6| Congenital nevus

|7| Reed/Spitz nevus |8| Melanoma |9| Other:…………………………….

Histopathological Diagnosis

|1| Solar Lentigo |2| Compound melanocytic nevus |3| Seborrheic keratosis

|4| Lentigo maligna |5| Dermal melanocytic nevus |6| Melanoma in situ

|7| Dysplastic nevus |8| Invasive Melanoma |9| Others:…………………………….

Breslow thickness |1| ≤1 mm |2| 1-2 mm |3| 2 -4 mm |4| >4 mm
